# Combining Optical Character Recognition With Paper ECG Digitization

**DOI:** 10.1109/JTEHM.2021.3083482

**Published:** 2021-05-25

**Authors:** Shambavi Ganesh, Pamela T. Bhatti, Mhmtjamil Alkhalaf, Shishir Gupta, Amit J. Shah, Srini Tridandapani

**Affiliations:** 1 School of Electrical and Computer EngineeringGeorgia Institute of Technology 1372 AtlantaGA30332USA; 2 Department of EpidemiologyRollins School of Public HealthEmory University 1371 AtlantaGA30322USA; 3 Department of RadiologyUniversity of Alabama at Birmingham 9968 BirminghamAL35249USA

**Keywords:** Electrocardiography, electronic medical record, optical character recognition, connected component analysis

## Abstract

Objective: We propose a MATLAB-based tool to convert electrocardiography (ECG) waveforms from paper-based ECG records into digitized ECG signals that is vendor-agnostic. The tool is packaged as an open source standalone graphical user interface (GUI) based application. Methods and procedures: To reach this objective we: (1) preprocess the ECG records, which includes skew correction, background grid removal and linear filtering; (2) segment ECG signals using Connected Components Analysis (CCA); (3) implement Optical Character Recognition (OCR) for removal of overlapping ECG lead characters and for interfacing of patients’ demographic information with their research records or their electronic medical record (EMR). The ECG digitization results are validated through a reader study where clinically salient features, such as intervals of QRST complex, between the paper ECG records and the digitized ECG records are compared. Results: Comparison of clinically important features between the paper-based ECG records and the digitized ECG signals, reveals intra- and inter-observer correlations of 0.86–0.99 and 0.79–0.94, respectively. The kappa statistic was found to average at 0.86 and 0.72 for intra- and inter-observer correlations, respectively. Conclusion: The clinically salient features of the ECG waveforms such as the intervals of QRST complex, are preserved during the digitization procedure. Clinical and Healthcare Impact: This open-source digitization tool can be used as a research resource to digitize paper ECG records thereby enabling development of new prediction algorithms to risk stratify individuals with cardiovascular disease, and/or allow for development of ECG-based cardiovascular diagnoses relying upon automated digital algorithms.

## I. Introduction

Electrocardiography (ECG) is a diagnostic tool used by medical professionals to record the electrical activities of the heart [1], and is a critical tool for determining abnormalities of the heart such as cardiac arrhythmias, coronary heart disease, congenital heart defects, and myocardial infarction [2]. Supported by ongoing technological advances, patient information is efficiently digitized and stored in their Electronic Medical record (EMR) [3]. However, while ECG records are increasingly stored in EMRs using digital formats, legacy systems require that ECG records largely continue to be printed on paper for analysis. Therefore, automated retrospective analysis of ECG records at scale is difficult unless the paper records are preserved carefully and digitized accordingly [4]. Importantly, digitized ECG records can be utilized by prediction algorithms for further analysis of clinical parameters, and can potentially help contribute to the advancement of current knowledge on clinical diseases [5], [6].

There have been attempts to standardize ECG records, and as summarized in the review paper by Waits and Soliman [7], groups such as the American College of Cardiology Foundation [8] and the International Society for Computerized Electrocardiography [9] have moved to digitize the ECG records independently of ECG vendors. However, there is no clear consensus about a universal standard format to store ECG records exemplified by the ECG records produced by the variety of systems in use [10]. Given the existence of vendor-specific and proprietary digital ECG records, there remains a pressing need to digitize ECGs consistently and easily. Thus, to account for the variety of formats available, a vendor-agnostic ECG digitization tool is required.

**FIGURE 1.**
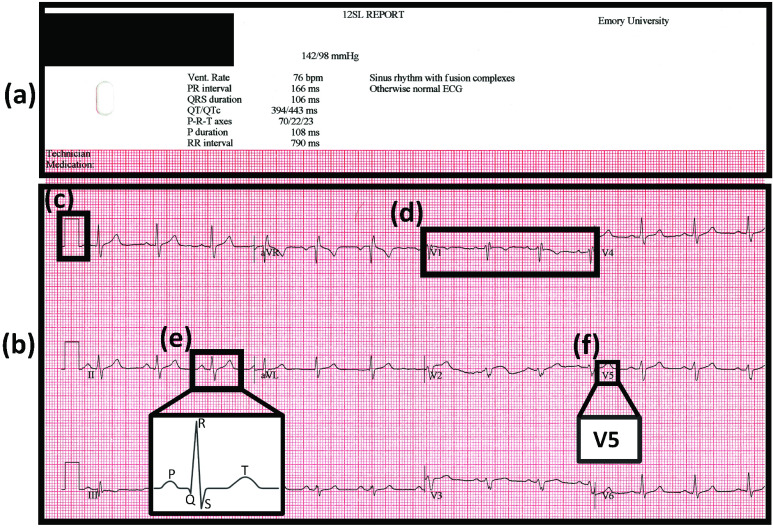
A representative ECG record, template, is displayed. (a) The patient demographic information is highlighted in the upper portion of the ECG record. (b) The ECG signal information is contained in the lower portion of the ECG record. (c) The ECG segments are preceded by a DC pulse, for calibration purposes. (d) A typical ECG segment consists of 3 consecutive heart beats. (e) Each heartbeat is characterized by the peaks P, Q, R, S, and T. (f) For identification purposes each ECG segment has an associated ECG lead character name (e.g. V5). Note, all identifying patient information has been redacted.

To advance the digitization of ECGs, we build upon our previous work [11], [12] and tackle one of the main challenges in digitizing paper records: digitization of signal which overlaps with printed characters that identify the lead (Figure 1). This overlap interferes with accurate digitization. Existing methods utilize morphological processing and connected component analysis (CCA) [13] for removal of non-overlapping characters [14], but the extent to which overlapping ECG characters affects the digitization process has not been addressed and thoroughly investigated. While CCA does detect and categorize isolated characters, overlapping characters are often still categorized to be a part of the original wave, making it harder to remove them.

We propose the use of Optical Character Recognition (OCR) [15] for removal of overlapping ECG lead characters on the ECG waveforms. OCR allows for identification of characters within the ECG records, thereby separating the characters that overlap the waveform. This helps minimize the distortion of the waveform. Apart from removal of ECG lead characters, OCR also aids in extracting patient information contained in the ECG records, to link them to the corresponding research record or the EMR.

In addition, we present a semi-automated segmentation procedure using a method based on CCA to extract ECG row waveforms in the records. Moreover, the proposed CCA partly aids in character removal by discarding non-overlapping characters. While previous studies utilize a connected components based approach to extract ECG lead segments [14], [16], in the current work we employ CCA along with a distance-based metric to allocate the connected components to the appropriate ECG row waveforms. Although automatic segmentation strategies for ECG waveforms exist [16], these strategies are restricted because they assume rigid placement of signals from various ECG leads within the paper record. The region containing each waveform is detected based on bounding boxes, which may lead to inaccurate digitization in instances wherein the signals overlap with one another. Other proposed approaches follow similar procedures and can only digitize records containing 12 lead segments [17], [18]. An additional challenge is baseline drift. Our CCA based approach in this paper attempts to segment the ECG row waveforms without explicitly computing the bounding boxes or segmentation markers for ECG leads and therefore it can accommodate such drift.

The second barrier is the lack of open accessible software for digitization of ECG records. This is hampered by the lack of generalizability across vendors of digital ECG systems [16], [19], and limited availability to researchers [19], [20] due to their proprietary nature. While validated open source applications exist for extraction of ECG waveforms from ECG records stored in PDF format [21], [22], these applications do not extend to digitization of scanned stored images. With respect to mobile platforms, an Android application [23] has been developed but has not been clinically validated. Furthermore, there is limited statistical evidence to confirm the robustness of these approaches, thereby limiting the generalizability of the methods over vendor-dependent ECG records.

To address this issue, vendor-specific information is bounded by a template. For example, ECG templates contain characteristic information, such as patients demographics (Figure 1(a)), the location of ECG waveforms (Figure 1(b)), ECG lead characters (Figure 1(f)), color of the ECG record and DC pulses (Figure 1(c)). While utilizing a purely template-based approach fails to accurately digitize ECG records containing erratic ECG signals [12], our hybrid combination of template- and CCA-based methods allows for greater flexibility while segmenting ECG waveforms. Once the template information is provided, the row waveforms can be segmented regardless of the number of ECG lead segments in the record.

The culmination of our effort is the realization of a tool for digitizing paper ECGs as an open source standalone executable tool with a graphical user interface (GUI) designed in MATLAB (MathWorks: Natick, MA). To support the utility of the tool, ECG digitization results are validated through a reader study conducted at Emory University Hospital. Cardiologists (observers) exercised the tool to measure the correlation of the clinical features between the digitized ECG record and the paper ECG record using a kappa coefficient [24], as well as intra- and inter-observer statistics.

The remainder of the paper is organized as follows. Section II details the methods involved in the digitization of the ECG records, and validation techniques used in the reader study and the results obtained from the validation study are presented in Section III. Section IV discusses these results, and Section V presents the conclusion.

## II. Methodology

This section describes the process of converting scanned paper ECG records in JPEG (Joint Photographic Experts Group) format into digitized ECG signals. Methods of optical character recognition (OCR) to extract patient demographic information and removal of ECG lead characters are also detailed.

### A. ECG Digitization and OCR Overview

**FIGURE 2.**
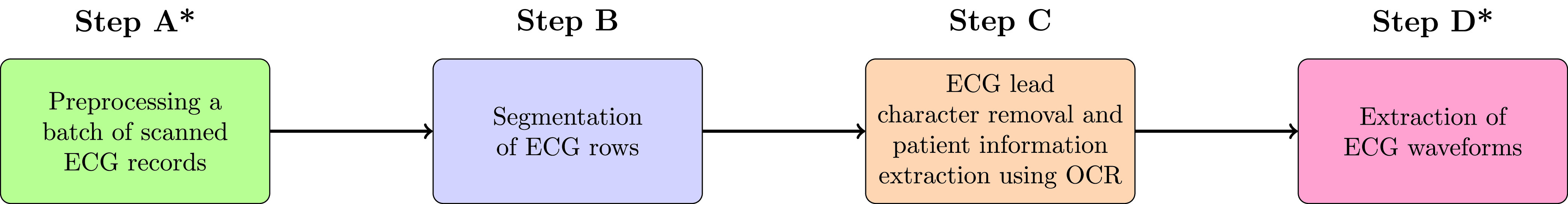
Flowchart depicting the high-level steps of the ECG digitization process, in which the fully automated steps, Step A and Step D, are marked by an asterisk.

**FIGURE 3.**
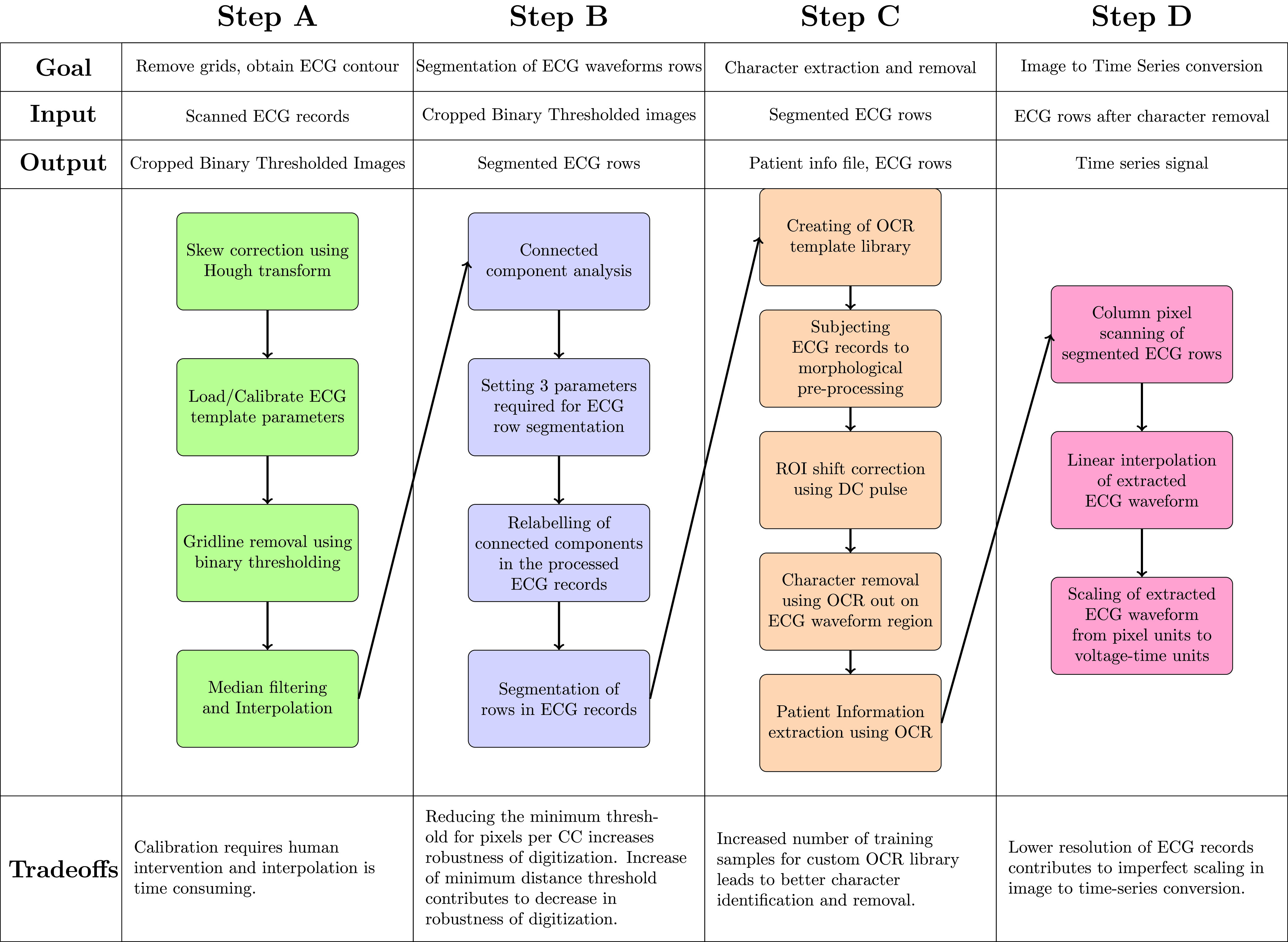
The extended version of ECG digitization flowchart, elaborating on the algorithms implemented and highlighting the tradeoffs for the methods chosen in each step, with respect to accuracy and time complexity for digitization.

A typical ECG record is comprised of 12 ECG leads, superimposed on a background grid for ease of readability and clinical feature measurement (Figure 1). The process of ECG signal digitization involves the conversion of a 2-Dimensional image to a 1-Dimensional signal, wherein each point is specified as a voltage-time pair. In addition, OCR removes ECG lead characters from the image, and is used to extract patient information. The overall digitization process is depicted by a simple flowchart (Figure 2), with each of the four steps (A-D) detailed in the following subsections, along with an associated validation procedure (Figure 3). Additionally, an associated validation procedure is described in Step E.

### B. Step A: Preprocessing of Scanned ECG Records

**FIGURE 4.**
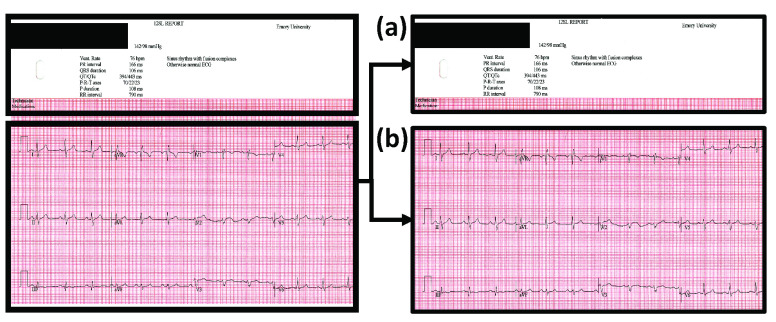
This figure illustrates the partitioning of an ECG record based on the patient demographic information and ECG waveform information with a representative scanned ECG record on the left as the starting point. (a) The partitioned patient demographic information region of the ECG record. (b) The partitioned ECG waveform region of the ECG record.

The preprocessing functions for a batch of scanned ECG records are described in the blocks of Step A (Figure 3). The scanned paper ECG records are initially converted into grayscale 8-bit images. The original ECG records can either be preserved at their original resolution or downscaled to 300 DPI resolution to reduce computation time of the algorithm. Due to human error, paper ECG records are often scanned at a skewed angle. To rectify this, the Hough Transform [25] is used to identify the angle of skew using the gridlines in the background of each of the ECG records individually. The scanned ECG records are thus reoriented using the obtained angle of skew. Following this, the portion of the scanned ECG records containing ECG signals are segregated from the portion of the scanned ECG records containing patient demographic information (Figure 4).

Next, an automatic thresholding technique utilizing the histogram profile of the background gridlines is carried out on the portion of the ECG records containing the waveforms, based on our prior work [11]. Although this is a suitable technique to apply to most scanned ECG records, there may be a requirement for additional manual intervention in instances wherein the ECG records are faded with faint ECG signals. In such cases, an option to modify the threshold value is included. The thresholding technique results in the removal of the background grid in the binarized thresholded ECG records. Due to this, salt and pepper noise is sometimes introduced and can be removed using a $3\times 3$ median filter. Aside from the noise, discontinuities in the ECG signals may result from erosion of pixels by thresholding. These gaps can be bridged using linear filtering on the binarized ECG records. Thus, the removal of the background gridlines and conversion of scanned ECG records to binarized ECG records is achieved in Step A.

### C. Step B: Segmentation of ECG Row Waveforms

**FIGURE 5.**
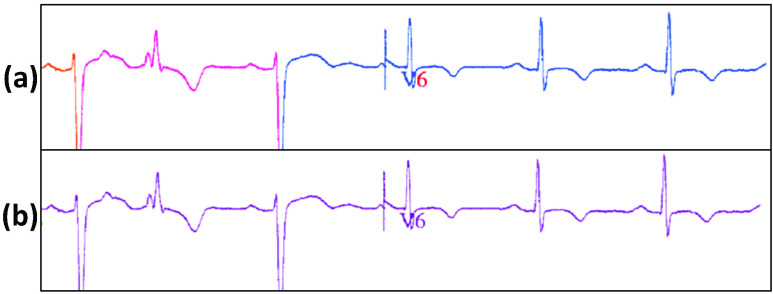
CCA is applied to the binarized ECG record and the resulting connected components are allocated to their respective ECG waveform segments. (a) Multiple connected components are identified due to discontinuities in the ECG row waveform, each of which are identified by a different color in the waveform. (b) The multiple connected components are now identified and assigned to their original row waveform.

Step B of the flowchart contains blocks describing the segmentation of ECG row waveforms in the binarized ECG records, using a CCA based method (Figure 3). The binarized ECG records containing the ECG row waveforms are provided as input in Step B. Contiguous regions, known as connected components, are obtained in a binary image using a predetermined heuristic for connectivity between pixels. The pixels of an identified connected component region are uniquely labeled using connected component labelling (CCL). Upon subjecting the binarized ECG records to this method, the uniquely labelled pixels corresponding to the connected component regions are obtained (Figure 5(a)). CCA based method of the binarized ECG records and labeling of pixels in connected components are carried out by built-in MATLAB functions, such as *bwconncomp* and *regionprops*, respectively. Subsequently, the CCA based method results in a number of labelled connected components which need to be combined and reorganized into ECG row waveforms.

The reorganization of the ECG waveforms is complicated by the discontinuities in the ECG waveforms, which are in part attributed to thresholding and the extent of degradation of the ECG records. To overcome this, connected components within a fixed distance threshold are fused together. This distance threshold is set such that all the connected components belonging to the same ECG row waveform are combined. Having too large a threshold may lead to inclusion of stray pixels near the ECG signal, which may introduce distortions in the digitized ECG waveforms.

The choice of the distance metric determines the computational efficiency of the digitization process. For example, using Hausdorff distance [26] where all the pixels in the connected components are considered for the distance measurement, takes $O(N^{2})$ computations, where N is the number of pixels in the components. If N is a large number, the time taken for digitization can be very large. Instead a modified version of the Hausdroff distance metric is implemented such that only a select number of pixels at the starting and the ending portions of the connected components are considered. This is found to reduce computational time for the digitization process. Of note, choosing a smaller number of pixels may reduce the accuracy of the distance computed.

To further increase the computational efficiency of the digitization process, connected components whose number of pixels is lesser than a threshold, which is typically around 100 pixels, can be discarded. Inclusivity of a greater number of connected components lead to smoother ECG waveforms, ultimately leading to increased time for digitization of ECG records.

Thus, the following three main parameters can be modified to efficiently identify connected components comprising ECG row waveforms: (1) the threshold distance between each of the connected components, (2) the metric chosen for distance computation between the connected components, and (3) the number of connected components retained for distance computation. These parameters are chosen to optimize digitization accuracy, and the connected components are designated to their corresponding ECG row waveforms (Figure 5(b)). The ECG row waveforms are segmented using the connected components method in Step B.

### D. Step C: Character Removal and Patient Information Extraction Using OCR

Step C utilizes optical character recognition (OCR) to identify characters to extract patient demographic data, and to removal of overlapping ECG lead characters. OCR utilizes correlation as a metric to identify text, using a predefined template-based library. MATLAB contains a built-in OCR application which facilitates the creation of custom OCR template-based libraries. The OCR application in MATLAB contains a predefined library of numeric characters (Zero - Nine), and the lower-upper cases of the English alphabet. The OCR application also provides the option of creating custom libraries which are created using character extraction, training, and annotation on sample records. Manual annotation of characters is necessary sometimes due to misidentification of characters by the OCR software.

**FIGURE 6.**
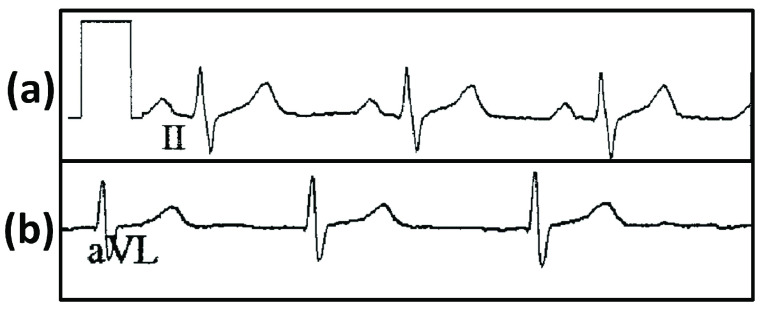
The ECG records after thresholding and grid removal are displayed to illustrate the issue of overlapping text on ECG signals. (a) The ECG lead character ‘II’ is well separated from the ECG signal. (b) The ECG lead character ‘aVL’ overlaps the ECG signal.

The binarized ECG records obtained from Step A are then subjected to morphological operators such as erosion, followed by dilation. This clarifies the underlying skeletal structure of the characters. OCR is utilized to identify and remove the overlapping ECG lead characters to ensure only the ECG waveforms are present (Figure 6). The ROI regions corresponding to the ECG lead characters are designated manually by the user for each type of template. These ROI regions are the boundaries within which OCR is carried out. Thus, character removal of ECG lead characters is achieved. To smoothen the abrupt transition between signals at the locations of character removal, linear filtering is applied.

Following this, the patient demographic information present in the ECG records is extracted using OCR and may be linked to the patient’s research records or EMR. The extracted characters are stored in a text file or in the header information and available to the user.

However, with respect to character removal of ECG lead characters, occasionally a few characters are only partially identified and removed. The cause has been traced to imperfect alignment of scanned ECG records due to human error. The ROIs are shifted around, and character removal is sub-optimal for these ECG records. To circumvent this, the scanned ECG records are compared with standard templates, which are the original scanned paper ECG records without anomalies either obtained from a public repository or selected by the user from the available records. The difference in alignment is addressed by adding the offset to the ROIs. Thus, using OCR, the overlapping ECG lead characters are removed.

### E. Step D: Extraction of ECG Waveforms

The segmented ECG row waveforms, obtained from Step B, are extracted and converted to voltage-time pairs in Step D (Figure 3). The columns of the binarized image are scanned and the coordinates of the pixels are stored. Due to the thickness of the ECG signals printed on paper ECG records, an ECG waveform in the binary image also has a thickness associated with it. Therefore, in a given column, the mean of the row indices are utilized for conversion to the time-series signal. Linear filtering and interpolation are implemented to bridge the discontinuities in the waveforms. The time-voltage pairs are obtained for the contours of the ECG signal, and the scanned ECG record is digitized.

### F. Step E: Validation Procedure

**FIGURE 7.**
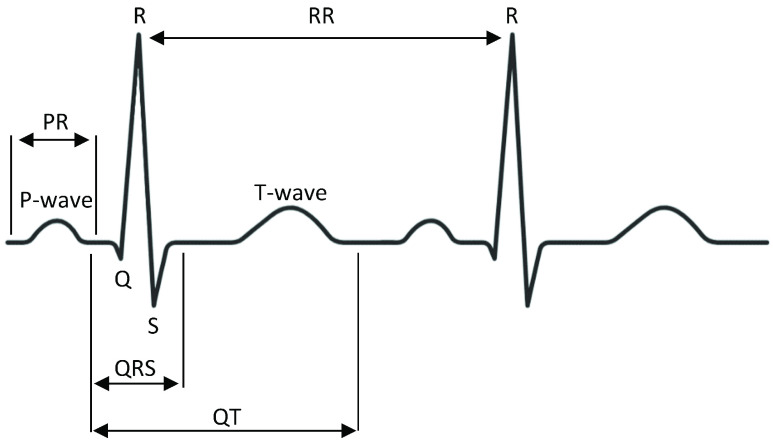
Four clinical features of a typical ECG lead segment are illustrated, namely *PR* interval, *QRS* interval, *QT* interval, and *RR* interval.

To confirm the quality of digitization of paper ECG records, we conducted a reader study identifying five clinically important features of the ECG signal. The features included the $PR$ interval (lead II), $QRS$ interval (lead V1), $QT$ interval (lead V3), $RR$ interval (lead V6) and the $QT_{C}$ interval (Figure 7). The $QT_{C}$ interval is formulated using the equation $QT_{C} = \frac {QT}{\sqrt {RR}}$. $QT_{C}$ prolongation ($QT_{c} > 450$ ms), first-degree atrioventricular block ($PR > 200$ ms), bradycardia ($RR > 1$ sec), and intra-ventricular conduction delay ($QRS > 120$ ms) were calculated to verify the validity of the digitization process. Based on these values, a kappa statistic was calculated to measure the agreement that occurs beyond that of chance.

The study was carried out by two cardiology observers, Observer A and Observer B with 2 and 4 years of experience in interpreting ECGs, respectively, to calculate the inter-observer and the intra-observer measurements. For the purpose of this study, the intra-observer measurements are defined as the scanned paper and digital ECG measurements obtained by the observer on 2 separate days. A total of 10 ECGs randomly selected from a subset of 53 ECG records were examined retrospectively upon approval from the Emory Institutional Review Board (IRB00086259). Five parameters from 10 printed scanned ECG records and 10 digitized ECG records were measured by the two cardiology observers in two-interpretation sessions, between which there was 1-week of washout period. During the 1^st^ interpretation session, cardiology observer A measured the 5 parameters from the odd-numbered standard ECG records, and even-numbered digitized ECG records. Cardiology observer B measured the 5 parameters for the odd-numbered digitized ECG records, and even-numbered standard ECG records. During the second interpretation session, the observers interpreted the remaining ECG records from the two sets. Upon the completion of the study, the results were tabulated, and pearson correlation coefficients computed.

## III. Results

This section details the results obtained from the ECG digitization process and the validation study.

### A. ECG Digitization Process

**FIGURE 8.**
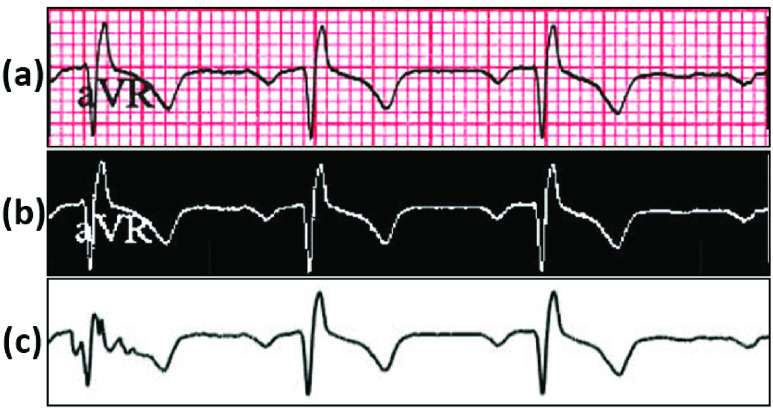
The intermediate results are shown sequentially for the ECG digitization process. (a) Sample paper ECG record without any processing; (b) Image obtained after application of thresholding and filtering; (c) Digitization of ECG segment without ECG lead character removal using column pixel scanning.

**FIGURE 9.**
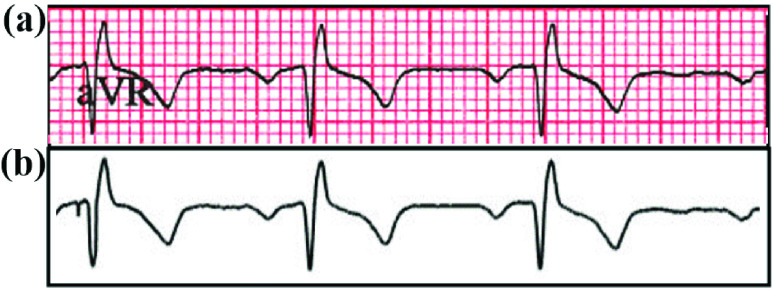
Digitization of scanned paper ECG samples with the inclusion of character removal. (a) The original ECG waveform is displayed containing the ECG lead character “aVR”. (b) ECG waveform is digitized, with the ECG lead character ‘aVR’ removed during the process of digitization.

Figure 8 details the intermediate stages of the process of ECG digitization process. Beginning with a paper ECG record (Figure 8(a)), the image is thresholded and filtered to extract the ECG trace (Figure 8(b)). In the final step, digitization of the ECG segment is carried out initially without ECG lead character removal (figure 8(c)). Afterwards, the digitization is implemented with ECG lead character removal (figure 9).

### B. Validation Study

Five clinically important features, namely $PR$ interval, $QRS$ interval, $QT$ interval, $RR$ interval and $QT_{c}$ interval, of the ECG signals were identified by 2 observers (observer A and observer B) and the results of the inter-observer and the intra-observer correlations of the clinical parameters are presented in Table 1. Except for the $QT_{c}$ interval of the inter-observer measurements, the correlations calculated are found to be in the range 0.80 – 0.99. This indicates that the clinical parameters are preserved during the process of digitization of scanned ECG papers. The kappa statistic averages at 0.86 for intra-observer correlations, and 0.72 for the inter-observer correlations. Thus, the desired clinical parameters are preserved during digitization.

**TABLE 1:** Intra-Observer and Inter-Observer Correlations for the 5 ECG Parameters

Parameter	Correlation Coefficient, (Intra-observer A)	Correlation Coefficient, (Intra-observer B)	Correlation Coefficient, (Intra-observer)
$PR$	0.9732	0.9711	0.9310
$QRS$	0.9628	0.9974	0.9438
$QT$	0.9046	0.9623	0.8115
$RR$	0.8830	0.9285	0.8360
$QT_{c}$	0.8653	0.8942	0.7954

### C. OCR

Apart from the results of the reader study, the OCR application successfully obtained the text of the patient demographic data contained in the paper ECG record such as the name of the university, patient ID, date, time, and clinical parameter details. The text is obtained with an accuracy of 93.56% which is expected to increase with the inclusion of more training samples for the OCR template library.

### D. Tradeoffs

Table 2 provides the tradeoffs relating to the time taken to digitize a single scanned paper ECG record over a range of parameter values. As detailed in the methodology section, the digitization process is sensitive to three parameters: (1) the threshold distance between each of the connected components, (2) the metric chosen for distance computation between the connected components, and (3) the number of connected components retained for distance computation. The simulations were run on a device with Intel(R) Core (TM) i7 processor with a clock speed of 2.3Ghz and 16GB RAM. This is done with respect to single template of an ECG record, of a particular vendor. The number of pixels considered for distance computation varies between 50 to 100, resulting in a range of values shown in the table. Using this table as a rough guide, the appropriate parameters can be chosen for the digitization of ECG records.

**TABLE 2:** Time Taken (In Seconds) for Digitization of an ECG Record Over a Range of Parameter Values

	Minimum Number of Components Chosen for CCA		
Distance Threshold	50	500	800
10	43.91–62.02	39.79–57.11	20.93–25.87
500	44.87–64.83	41.91–58.39	20.84–25.64
5000	47.62–68.31	40.73–62.21	22.39- 27.41

## IV. Discussion

The aim of this work was to extract the ECG signals and produce a digitized format from the scanned ECG records, and consequently create an open source standalone executable tool that is independent of equipment vendor. Importantly, the correlations obtained in the reader study results indicate that the process of digitization of ECG records preserves the critical clinical parameters of ECG signals. Furthermore, the results of the reader study also corroborate the results put forth by our previous study which show high correlation of clinical data extracted from paper and digitized ECG formats [11].

Although most correlations were > 0.9, some of them were slightly lower. Discrepancies remain in the correlations between the paper ECG signal in the paper and digitized records, which we hypothesize can be attributed to differences in the thickness of the ink used to print the ECG signal. Furthermore, small differences in the sampling rate of the signals between the paper and digitized records, can also contribute to imperfections in correlations. The relatively lower correlation values associated with the inter-observer values of the $QT_{c}$ interval can be explained by the differences in the technique used, such as the estimation of the beginning and end points of an ECG lead segment, used by the observers in the study for the $QT$ and $RR$ interval. This set of discrepancies was further compounded and carried over due to the calculation required for the $QT_{c}$ interval. Additionally, we observed that most of the error occurs in extraction of patient demographic information using OCR occurs when the text overlaps with the background grid in the ECG. The binary threshold used in the OCR process differs from that of the threshold used during extraction of the ECG waveforms, due to the presence of the background grid. Therefore, the text overlapping the grid may not be correctly binarized due to the varying threshold requirements.

The main contributions of this work include the removal of overlapping ECG character lead segments using morphological processing and OCR, segmentation of ECG row waveforms using CCA and batch processing of ECG records which can be generalized across manufacturers of ECG systems. Primarily, in the removal of overlapping ECG character lead segments, the utilization of OCR and morphological processing allows for accurate demarcation between the ECG signal and ECG lead characters. Based on the reader study results, OCR can identify the remnants of a lead character fused with that of a signal, while ensuring that the vital features of the ECG signal such as $PR$ interval, $QRS$ interval, $QT$ interval, $RR$ interval and $QT_{c}$ interval still remain preserved. Based on our previous work, OCR is also utilized for the extraction of patient demographic information from ECG records, to ultimately link it to the patient’s research record or EMR [11].

Another contribution of this work is the segmentation of the ECG waveforms using a connected components based method. This approach is designed to ensure maximum retention of the saliency of clinical features previously mentioned. An additional advantage is character removal. That is, a significant portion of the ECG lead characters that do not bleed into the ECG signals are discarded, since they fall into the category of connected components, which contain too few pixels. Additionally, during the process of combining the connected components to form ECG row waveforms, the components which are deemed to be at a distance greater that the threshold are discarded. Therefore, stray non-overlapping ECG lead characters identified to be a connected component are often removed. This aids OCR in discarding ECG character leads overlapping with the ECG signals.

Batch processing of ECG records is another key feature in this work. A one-time creation of template corresponding to each unique type of ECG record is necessary, to store information regarding the location of ECG contours, patient demographic information, and ECG lead characters. These templates facilitate the batch processing of multiple ECG records, to convert them into digitized records. An online library of publicly available ECG templates, along with a user manual and the application installation files, will be available on Github at https://github.com/ECG-Digitization-Project.

Finally, this work has been packaged into a standalone executable tool, available to the public. The tool was designed utilizing ‘App designer’ available on MATLAB R2019b. This application was designed utilizing ‘App designer’ available on MATLAB R2019b and also incorporates Multi ROI/Mask Editor Class package [27] designed using the same. The algorithm is executed in a modular manner, for the purpose of easier modifications and to allow easy implementation of different methods in the future. The installation instructions, along with the manual for the ECG tool is presented in the supplementary section.

With regards to tradeoffs in the methods, the quality of ECG digitization using this work is dependent on the parameters chosen in the CCA. These parameters can be modified for a template, to control the trade-offs between the time taken for digitization and quality of digitized ECG records Table 2. The time values presented in the table denote the total time taken for digitization once the parameters are established. This is very heavily dependent on factors such as degradation of the ECG record, and quality of the ink used for print ECG signals. It should be noted that for most of the previous work mentioned thus far, time required for digitization of ECG records has not been thoroughly investigated.

ECG records that are of relatively lower quality due to graininess of the paper require the parameter corresponding to the minimum number of pixels selected per connected component to be also lower. In contrast, higher quality ECG records containing smoother ECG signals require the parameter to have a higher number of pixels as the threshold. This would prolong the digitization time for grainy ECG records, but would also ensure the thorough inclusion of all the signal components. Similarly, the distance computed between each of the connected components helps determine the ECG waveform to which the connected components belong. The larger the distance computed,the more likely it is for the ECG waveform to include stray pixels that do not belong to the signal. Conversely, the smaller the distance computed, the slower the digitization with increased robustness.

A limitation of utilizing inter-observer and intra-observer correlations is the introduction of bias, as observed in our earlier work [11]. A week of washout period helps in minimizing the bias of intra-observer correlation to an extent, but may still be present. Variations in the approach and judgment of waveform features affect the inter-observer correlation calculations. This leads to underestimation of correlation coefficient to a significant extent. Another opportunity exists to understand the impact of lead interpolation on the ECG digitization tool, more specifically, when fewer than 12 leads are applied to the patient but interpolated to a 12-Lead ECG.

While manual intervention is required, such as entering the ECG template-based information and calibration, once the template information is entered, the user can continue to use the tool for that batch of records. Additionally, the template approach for the segregation of the patient demographic information and ECG waveform region, inherently assumes the presence of a DC pulse at the beginning of ECG lead segments. Thus, the recalibration of the parameters with respect to the template ECG records will now be more difficult to implement in the absence of a DC pulse. Another limitation of the proposed work is the difficulty in digitizing overlapping ECG waveforms across consecutive ECG rows, in the presence of high signal amplitudes. Using the current approach, the separation of the overlapping waveforms remains to be addressed.

Furthermore, use of the ECG digitization tool may result in reduced accuracy of waveform tracings in areas that overlap with text. This may lead to misclassification of beat type (normal versus ventricular ectopic), and further testing is needed on the impact of this signal deformation on computerized outcomes of programs that analyze the signal. Finally, due to limited ECG records available for the construction of the custom library, the OCR module did not contain many template characters. With the availability of more records, the OCR process will result in higher accuracy of character recognition and extraction. An additional disadvantage is that some portions of the waveform may be altered due to OCR error. This is also one of the current limitations of the algorithm.

The proposed digitization method can be improved in several ways in the future. For instance, subtractive clustering techniques and principal component analysis can be utilized for the separation of overlapping high amplitude waveforms [28]. Since this is an open sourced tool, the collection of ECG templates can steadily increase based on usage, which in turn can greatly improve the flexibility provided by the ECG tool. Similarly, the OCR template-based library can grow to be more robust as more ECG samples are utilized for the tool. Moreover, to further aid in the smoothing process of the ECG waveforms post-character removal, techniques such as inpainting [29] or bilateral filtering [30] approaches can be used.

## V. Conclusion

We developed an open-source MATLAB-based GUI tool for the conversion of paper ECG records to digitized ECG waveforms. The important features of this method include the use of OCR to perform character removal of ECG lead characters, and a tool to improve the generalizability of ECG record digitization regardless of the ECG vendor. The validation study conducted revealed negligible differences between the clinical features, such as $PR$ interval, $QRS$ interval, $QT$ interval, $RR$ interval and $QT_{c}$ interval of the paper-based and digitized ECG signals. Furthermore, the tool is manufacturer agnostic. More specifically, OCR character removal and digitization methods can be applied to paper records from any manufacturer. Subsequently, retrospective studies conducted using this tool can provide the medical community with information that can be used to discover ECG features of great prognostic value, along with advancement of knowledge in the domain of cardiac diseases.
